# Single-molecule real-time (SMRT) sequencing technology shows advantages in the clinical diagnosis of complex thalassemia

**DOI:** 10.3389/fmed.2026.1833379

**Published:** 2026-10-19

**Authors:** Ning Tang, Shiqiang Luo, Xingyuan Chen, Jiwei Huang, Xiaoni Wei, Dejian Yuan, Qingyan Zhong, Aiping Mao, Di Cui, Zhong Lin, Dingyuan Zeng

**Affiliations:** 1 Liuzhou Hospital Division of Guangzhou Women and Children's Medical Center, Liuzhou, Guangxi, People's Republic of China; 2 The Reproductive Hospital of Guangxi Zhuang Autonomous Region, The Reproductive Health Research Center of Guangxi Zhuang Autonomous Region, Nanning, People's Republic of China; 3 Department of Laboratory Medicine, Guangxi Hospital Division of The First Affiliated Hospital, Sun Yat-sen University, Nanning, Guangxi, People's Republic of China; 4 Guangxi Health Commission Key Laboratory of Birth Cohort Study in Pregnant Women of Advanced Age, Liuzhou, Guangxi, People's Republic of China; 5 Guangxi Clinical Research Center for Obstetrics and Gynecology, Liuzhou, People's Republic of China; 6 Liuzhou Key Laboratory of Thalassemia Precision Prevention and Treatment, Liuzhou, Guangxi, People's Republic of China; 7 Liuzhou Key Laboratory of Birth Defects Prevention and Control, Liuzhou Maternity and Child Healthcare Hospital, Liuzhou, Guangxi, People's Republic of China; 8 Liuzhou Maternity and Child Healthcare Hospital, Liuzhou, Guangxi, People's Republic of China; 9 Berry Genomics Corporation, Beijing, People's Republic of China

**Keywords:** Single molecule real-time (SMRT) sequencing, Complex thalassemia, Complex structural variants, Genetic diagnosis, Molecular diagnosis

## Abstract

**Background:**

Thalassemia is a hereditary hemolytic anemia caused by pathogenic variants in the α- or β-globin genes, resulting in imbalanced globin chain synthesis. Clinical manifestations vary from asymptomatic carrier states to severe transfusion-dependent anemia. Current routine diagnostic methods in China, gap polymerase chain reaction (gap-PCR) and reverse dot blot hybridization (RDB), detect only 24 common variants, leading to frequent genotype-phenotype mismatches and undiagnosed cases.

**Results:**

In this study, we employed multiplex ligation-dependent probe amplification (MLPA), Sanger sequencing, and SMRT sequencing to genetically screen 30 cases of complex thalassemia, aiming to clarify the underlying genetic causes. SMRT sequencing successfully resolved 28/30 cases (93.3%), with genotypes confirmed by MLPA and Sanger sequencing. Notably, three novel deletions were identified: a 32.8-kb (--^32.8^, chr16:149,860–182,697) and a 5.9-kb (chr16:169,916–175,863) deletion in the α-globin gene cluster, and an 8.3-kb deletion (chr11:5,224,209–5,232,469) in the β-globin gene cluster. In addition to detecting rare single-nucleotide variants (SNVs) and large deletions, SMRT sequencing additionally identified complex structural variants that are often missed or difficult to characterize using MLPA and Sanger sequencing.

**Conclusions:**

Our findings demonstrate that SMRT sequencing offers superior resolution and diagnostic power for complex thalassemia and is a valuable tool for improving molecular diagnosis in challenging cases.

## Background

Thalassemia is a severe, potentially life-threatening genetic blood disorder and a major congenital disease affecting populations worldwide. Globally, approximately 1.5% of the population carries a β-thalassemia allele, while 5% are carriers of an α-thalassemia allele (1). The clinical presentation of thalassemia varies widely, ranging from asymptomatic carriers to individuals with mild or moderate anemia, severe transfusion-dependent anemia, or even perinatal lethality (2). A variety of molecular assays have been developed to detect α- and β-globin gene variants (3), including multiplex amplifiable probe hybridization (MAPH) (4), multiplex ligation-dependent probe amplification (MLPA) (5), real-time quantitative polymerase chain reaction (qPCR) (6), quantitative multiplex PCR of short fluorescent fragments (QMPSF) (7), and melting curve analysis (MCA) (8). Each has specific strengths and limitations for screening and diagnostic purposes. In China, the gap polymerase chain reaction (gap-PCR) and reverse dot-blot (RDB) hybridization techniques remain the standard methods for routine molecular diagnosis. These methods allow for the detection of four common α-globin gene deletions (--^SEA^/, -α^3.7^/, -α^4.2^/, --^THAI^/) and three non-deletion variants associated with α-globin deficiency (α^WS^α/, α^CS^α/, and α^QS^α/), along with 17 common β-globin gene variants in the Chinese population. MLPA has been increasingly adopted in large medical centers due to its high sensitivity and specificity. It enables simultaneous detection of copy number variations (CNVs) across more than 40 genomic targets in a single reaction (9). Some research teams have also applied array comparative genomic hybridization (aCGH) to detect copy number changes in the α-globin gene (10). However, its widespread application is limited by the high cost of instruments and consumables. More recently, next-generation sequencing (NGS) has been introduced for thalassemia genetic screening (11). NGS allows for the detection of both known and novel variants, offering high sensitivity, accuracy, and simple sample processing (12). However, even with all of these tests, overall diagnostic yield for thalassemia remains below 50%, leaving a substantial proportion of rare or atypical cases unresolved. This gap is largely attributed to limitations in detecting complex variants, such as tandem repeat expansions and structural rearrangements that are not readily identifiable by conventional methods. The emergence of long-read sequencing (LRS) has opened new avenues for uncovering pathogenic variants in previously undiagnosed genetic conditions (13–18), as it enables highly scalable, accurate, and efficient genotyping (19, 20). Single-molecule real-time (SMRT) sequencing is an LRS sequencing technology that eliminates PCR amplification, providing long-read lengths from several hundred base pairs to hundreds of kilobases. It offers high accuracy, no GC bias, and single-molecule resolution (21). These features enable comprehensive analysis of rare or complex variants in *HBA1*, *HBA2*, and *HBB* genes, thereby enhancing clinical diagnosis and minimizing the risk of undetected variants (22).

In this study, we employed MLPA, Sanger sequencing, and SMRT sequencing to genetically characterize 30 complex thalassemia cases, aiming to elucidate the genetic basis of their phenotypes. By comparing the diagnostic performance of these three methods, we evaluated the clinical utility of SMRT sequencing for thalassemia screening and its role in genotype-phenotype correlation analysis.

## Methods

### Study population

Routine genetic analysis of α- and β-thalassemia variants was performed in individuals with abnormal hematologic screening results. In this study, “complex thalassemia cases” were defined as those meeting any of the following criteria: (1) the genotype detected by routine methods (gap-PCR combined with RDB) was significantly inconsistent with the hematological phenotype, including anemia severity, red blood cell parameters, or abnormal hemoglobin electrophoresis bands; (2) unexpectedly sized amplification products or abnormal electrophoresis patterns were observed, suggesting the possible presence of rare or novel large fragment deletions/duplications; (3) MLPA or Sanger sequencing indicated structural variations, but their precise breakpoints or *cis*/*trans* configurations could not be definitively resolved; (4) no pathogenic variant was detected by routine testing, yet the hematological phenotype strongly suggested a clinical diagnosis of thalassemia. This definition ensured the inclusion of cases that were difficult to diagnose conclusively using traditional methods. Exclusion criteria were: (1) incomplete basic information; (2) presence of other hematologic diseases that could confound the interpretation of genotype-phenotype associations. Specifically, we excluded individuals with: (a) moderate-to-severe iron deficiency anemia; (b) severe vitamin B_12_ or folate deficiency; and (c) active hematological malignancies.

From January 2010 to August 2022, all 30 enrolled patients with complex and difficult thalassemia (12 males and 18 females; age range: 4–51 years; mean age: 30.87 ± 8.45 years) underwent MLPA, Sanger sequencing, and SMRT sequencing in parallel. Their hematologic profiles and genetic testing results were collected and analyzed. This study was approved by the Ethics Committee of Liuzhou Hospital Division of Guangzhou Women and Children's Medical Center. Written informed consent was obtained from all participants.

### Routine blood and Hb electrophoresis analysis

Complete blood counts (CBCs) were performed using a hematocrit analyzer (XE-2100; Sysmex, Kobe, Japan). Hemoglobin (Hb) analysis was performed using high-performance liquid chromatography (HPLC, Variant II; Bio-Rad, Hercules, CA, USA) to detect Hb F, Hb A_2_, Hb H, and abnormal Hb content. According to the manufacturer's specifications, the lower detection limit for Hb F is 0.4%; levels below this threshold are reported as below the detection limit. Participants were considered to have abnormal hematologic screening results if any of the following criteria were met: (1) male with red blood cell (RBC) beyond range 4.0–5.5 × 10^12^/L; (2) female with RBC beyond range 3.5–5.0 × 10^12^/L; (3) male with Hb < 130 g/L; (2) female with Hb < 115 g/L; (5) mean corpuscular volume (MCV) < 80 fL; (6) mean corpuscular hemoglobin (MCH) ≤ 27 pg; (7) Hb A_2_ level <2.5% or ≥3.5%; (8) Hb F level >3.5%; (9) presence of abnormal Hb (23).

### Genomic DNA extraction

Genomic DNA was extracted from peripheral blood samples using a nucleic acid extraction kit (Yishengtang, Shenzhen, People's Republic of China) and an automatic nucleic acid extractor (LabAid820; Xiamen Zhishan Biological Technology, Xiamen, China). DNA concentration and purity were assessed using a nucleic acid analyzer (ACT Gene ASP-2680; USA).

### Routine genetic analysis of α- and β-thalassemia variants

Four common α-thalassemia deletions (--^SEA^, -α^3.7^, -α^4.2^, and --^THAI^) were detected using gap-PCR. Three common non-deletional α-thalassemia variants, including Hb Quong Sze (Hb QS, *HBA2*: c.377T>C), Hb Constant Spring (Hb CS, *HBA2*: c.427T>C), and Hb Westmead (Hb WS, *HBA2*: c.369G>C), were identified using RDB. Seventeen common β-thalassemia variants, including -29 (A>G) (*HBB*: c.-79A>G), codons 41/42 (-TTCT) (*HBB*: c.126_129delCTTT), -30 (T>C) (*HBB*: c.-80T>C), -32 (C>A) (*HBB*: c.-82C>A), -28 (A>G) (*HBB*: c.-76A>G), codons 14/15 (+G) (*HBB*: c.45_46insG), IVSI-5 (G>C) (*HBB*: c.92+5G>C), codon 17 (A>T) (*HBB*: c.52A>T), codon 26 (or Hb E) (G>A) (*HBB*: c.79G>A), codons 27/28 (+C) (*HBB*: c.84_85insC), codon 31 (-C) (*HBB*: c.94delC), codon 43 (G>T) (*HBB*: c.130G>T), codons 71/72 (+A) (*HBB*: c. 216_217insA), IVS-I-1 (G>T) (*HBB*: c.92+1G>T), IVS-II-654 (C>T) (*HBB*: c.316–197C>T), CAP +1 (A>C) (*HBB*: c.-50A>C), and initiation codon (T>G) (*HBB*: c.2T>G), were screened using PCR and RDB assays.

### MLPA analysis

MLPA was performed using the P140 and P102 probe kits (MRC-Holland, The Netherlands) to detect CNVs in the α- and β-globin gene clusters, respectively. The P140 kit targets the α-globin gene cluster on chromosome 16, whereas the P102 kit targets the β-globin gene cluster on chromosome 11 (24, 25). Amplified products were separated via capillary electrophoresis on a 3500Dx genetic analyzer (Applied Biosystems, Foster City, CA, USA).

### Sanger sequencing of the α- and β-thalassemia genes

Three sets of primer pairs were designed for PCR amplification and sequencing of the α- and β-globin genes (*HBA1*, *HBA2* and *HBB*) (26). DNA sequencing was conducted using the Sanger dideoxy terminated sequencing method (ABI PRISM® 3130 automated sequencer; Applied Biosystems, Foster City, CA, USA). The reference sequence was NM_000517. PCR amplification was performed using 50 ng of genomic DNA and 20 pmol of each primer in a C1000 Thermal Cycler (Bio-Rad Laboratories, Hercules, CA, USA).

All procedures were carried out following the relevant guidelines and regulations.

### SMRT sequencing

SMRT sequencing was conducted by an external laboratory (Berry Genomics, Beijing) using the PacBio Sequel II platform (Pacific Biosciences, USA). As previously described (20), multiplex, long-range PCR was performed to amplify regions encompassing known structural variants, single nucleotide variants (SNVs), and insertions/deletions (indels) in the *HBB*, *HBA1*, and *HBA2* genes. After purification, end-repair, and ligation with double-barcode adapters at both ends, SMRTbell libraries were constructed using the Sequel Binding and Internal Control Kit 3.0 (Pacific Biosciences, USA) (19, 20, 27). The libraries were loaded onto SMRT sequencing cells (Pacific Biosciences, USA) and sequenced in cyclic consensus sequencing (CCS) mode to generate 10–25 subreads per molecule. Raw subreads were processed by the CCS (Pacific Biosciences) and *lima* (Pbbioconda package), aligned to the GRCh38 reference using pbmm2 (version 1.5.0) software. SNVs and small indels were called using FreeBayes software (version 1.3.4). Structural variants were identified based on primer pair patterns, read length, and reference alignments using the HbVar, Ithanet, and LOVD databases, as previously reported (19). Haplotype analysis was conducted using WhatsHap (version 0.18) and pbampliconclustering software.

### Statistical analysis

To evaluate the impact of different α-globin gene defect types on hematological parameters, 28 cases with genotypes confirmed by SMRT sequencing were divided into three groups based on their α-globin gene defect status: (1) the α^0^-thalassemia heterozygote group, carrying α^0^-deletion alleles (--^32.8^, --^SEA^, or --^27.3^, *n* = 10); (2) the α^+^-thalassemia heterozygote group, carrying α⁺-deletion alleles (-α^5.9^, -α^4.2^, -α^2.4^, or -α^3.7^, *n* = 4); and (3) the non-α-defect group (*n* = 14). Intergroup comparisons were performed using one-way ANOVA. All statistical analyses were conducted using GraphPad Prism 9.0.2. Statistical significance was set at *p < 0.05, **p < 0.01, ***p < 0.001, and ****p < 0.0001; ns indicates no significant difference.

## Results

### Comparison between SMRT sequencing and different PCR-based methods

All 30 cases were analyzed using gap-PCR, RDB, MLPA, Sanger sequencing, and SMRT sequencing. Thalassemia-associated variants were identified in 28 cases, comprising α-globin variants (14 cases, 50.0%), β/δ-globin variants (9 cases, 32.1%), and combined α- and β/δ-globin variants (5 cases, 17.9%) (Table 1). The detected variants included 11 deletions [--^SEA^, -α^3.7^, -α^4.2^, --^32.8^, -α^5.9^, -α^2.4^, --^27.3^, *HBB*: 8.3-kb deletion, Chinese ^G^γ(^A^γбβ)^0^-thal, SEA-HPFH, Taiwanese deletion], one duplication (ααα^anti3.7^), four complex structural variants (HKαα, anti-HKαα, *HBA* fusion genes, Hb anti-Lepore LiuZhou), and five SNVs/small indels (*HBA1*: c.300+55G>T, *HBA2*: c.300+55T>G, *HBA2*: c.301-24delinsCTCGGCCC, *HBB*: c.315+5G>C, *HBD*: c.-127T>C). Notably, conventional gap-PCR and RDB could only detect three variants --^SEA^, -α^3.7^, and -α^4.2^. MLPA could indicate most structural variants but failed to resolve breakpoints for rare and novel deletions (e.g., --^32.8^, SEA-HPFH) or distinguish *cis*/*trans* configurations. Additionally, MLPA misclassified SNVs in cases 16–17 as small deletions. And the deletion of the α-fusion gene between *HBA2* and *HBAP1* was missed. Sanger sequencing identified all SNVs and small indels but could not determine *cis*/*trans* phasing in cases with multiple variants (cases 13–16), and tended to misclassify heterozygous variants with deletions as homozygous variants (cases 13–14). In contrast, SMRT sequencing accurately determined the genotypes in 28 cases, which were consistent with phenotypic presentations and supported by PCR-based methods. Therefore, SMRT sequencing achieved a diagnostic yield of 93.3% (28/30), significantly higher than that of conventional gap-PCR and RDB.

**Table 1:** Hematological phenotypes and genotypes of cases in this study.

		Hematologic screening									Routine genetic analysis of thalassemia (Gap-PCR and RDB)		MLPA		Sanger sequencing		SMRT	
Case	age (y) sex	RBC ×10^12^/L	Hb (g/L)	MCV (fL)	MCH (pg)	Hb A (%)	Hb A_2_ (%)	Hb F (%)	Hb H (%)	Abnormal Hb	α-globin gene cluster	β-globin gene cluster	α-globin gene cluster	β-globin gene cluster	α-globin gene cluster	β-globin gene cluster	α-globin gene cluster	β-globin gene cluster
1	35-F	5.74↑	120	65.4↓	20.9↓	86.2	2.8	0.4	—	—	ND	ND	One copy of the *HBZ*, *HBZP1*, *HBM*, *HBAP1*, *HBA2*, *HBA1* and *HBQ1* genes	ND	ND	ND	--^32.8^/αα	ND
2	36-M	5.3	153	87.6	28.8	97.7	2.3↓	—	—	—	ND	ND	One copy of the *HBA2* gene	ND	ND	ND	-α^5.9^/αα	ND
3	35-F	4.85	120	73.3↓	24.8↓	89.2	6.3↑	4.5↑	—	—	--^SEA^/αα	ND	--^SEA^/αα	One copy of the *HBB* gene	ND	ND	--^SEA^/αα	8.3-kb Del including the *HBB* gene, hete
4	51-F	4.85	118	73.4↓	24.3↓	92.1	6.9↑	1.0			ND	ND	ND	One copy of the *HBB* gene	ND	ND	ND	8.3-kb Del including the *HBB* gene, hete
5	28-F	5.2↑	124	77.3↓	23.8↓	70.3	16.5↑	0.6	—	Hb E?	ND	ND	ND	Three copies of part of the *HBB* and *HBD* genes	ND	ND	ND	Hb anti-Lepore Liuzhou, hete
6	4-F	5.43↑	113↓	62.9↓	20.8↓	45.6	14.2↑	0.6	—	Hb E?	--^SEA^/αα	ND	--^SEA^/αα	Three copies of part of the *HBB* and *HBD* genes	ND	ND	--^SEA^/αα	Hb anti-Lepore Liuzhou, hete
7	34-F	3.45↓	105↓	93.3	30.4	96	2.7	—	—	—	-α^3.7^/αα	ND	-α^3.7^/; ααα^anti4.2^/	ND	ND	ND	HKαα/αα	ND
8	32-M	4.66	124↓	82.6	27.8	97.2	2.5	—	—	—	-α^3.7^/αα	ND	-α^3.7^/; ααα^anti4.2^/	ND	ND	ND	HKαα/αα	ND
9	34-M	5.02	145	86	28.8	97.6	2.2↓	—	—	—	-α^4.2^/; -α3.7/; αα/	ND	-α^3.7^/αα	ND	ND	ND	-α^4.2^/HKαα	ND
10	29-M	5.98↑	141	72.1↓	23.6↓	97.9	2.1↓	—	—	—	--^SEA^/; -α^3.7^/; αα/	ND	--^SEA^/; -α^3.7^/; ααα^anti4.2^/	ND	ND	ND	--^SEA^/HKαα	ND
11	45-M	6.33↑	141	69.6↓	22.2↓	85.2	2.6	—	—	—	--^SEA^/; -α^3.7^/; αα/	ND	--^SEA^/; -α^3.7^/; ααα^anti4.2^/	ND	ND	ND	--^SEA^/HKαα	ND
12	25-M	6.26↑	133	67.3↓	21.2↓	97.7	2.3↓	—	—	—	--^SEA^/; -α^4.2^/; αα/	ND	--^SEA^/; -α^4.2^/; ααα^anti3.7^/	ND	ND	ND	--^SEA^/anti-HKαα	ND
13	32-F	3.85	75↓	75.7↓	19.5↓	89.2	1.2↓	0.5	10.1↑	—	--^SEA^/αα	ND	--^SEA^/αα	ND	*HBA2*: Nine SNPs^a^, homo	ND	--^SEA^/Fusion between *HBA2* and *HBAP1* (Nine SNPs)	ND
14	24-F	3.81	86↓	76.7↓	21.5↓	81.2	0.8↓	0.5	17.5↑	—	--^SEA^/αα	ND	--^SEA^/αα	ND	*HBA2*: Nine SNPs^a^, homo	ND	--^SEA^/Fusion between *HBA2* and *HBAP1* (Nine SNPs)	ND
15	28-M	3.86↓	101↓	77.7↓	23.6↓	73.1	1↓	—	21.8↑	—	--^SEA^/αα	ND	--^SEA^/αα	ND	*HBA2*: Nine SNPs^a^, homo	ND	--^SEA^/Fusion between *HBA2* and *HBAP1* (Nine SNPs)	ND
16	31-F	4.93	146	89.9	29.7	93.5	1.5↓	5↑	—	—	ND	ND	One copy of the *HBA2*-intr.2, three copies of the *HBA1*-intr.2	ND	*HBA2*: c.300 + 55T > G, hete *HBA2*: c.301-24delinsCTCGGCCC, hete	ND	*HBA2*: c.300 + 55T > G, hete *HBA2*: c.301-24delinsCTCGGCCC, hete	*HBD*: c.-127T > C, hete
17	24-F	4.75	120	78.8↓	25.3↓	97.3	2.6	—	—	—	ND	ND	One copy of the *HBA1*-intr.2, three copies of the *HBA2*-intr.2	ND	*HBA1*: c.300 + 55G > T, hete	ND	*HBA1*: c.300 + 55G > T, hete	ND
18	28-M	5.1	137	82.5	25.6↓	97	2.7	—	—	—	ND	ND	One copy of the *HBA1* gene	ND	ND	ND	-α^2.4^/αα	ND
19	28-F	6.21↑	130	65.6↓	21↓	97.7	2.3↓	—	—	—	ND	ND	One copy of the *HBZP1*, *HBM*, *HBAP1*, *HBA2*, *HBA1* and *HBQ1* genes	ND	ND	ND	--^27.3^/αα	ND
20	40-M	5.85↑	143	77.7↓	24.4↓	81.4	2.7	6.4↑	—	—	-α^3.7^/αα	ND	-α^3.7^/αα	Chinese I (*ε*γδβ)^0^, hete	ND	ND	-α^3.7^/αα	Chinese ^G^γ(^A^γбβ)^0^-thal, hete
21	29-M	5.22	142	81.4	27.3	65.7	2.8	31.5↑	—	—	ND	ND	ND	One copy of the *HBB* gene	ND	ND	ND	SEA-HPFH, hete
22	28-F	4.56	133	86.5	29.1	71	3.6↑	25.4↑	—	—	ND	ND	ND	One copy of the *HBB* gene	ND	ND	ND	SEA-HPFH, hete
23	38-F	4.22	109↓	72.2↓	23.8↓	72.8	4↑	23.2↑	—	—	ND	ND	ND	One copy of the *HBB* gene	ND	ND	ND	SEA-HPFH, hete
24	21-F	3.68	121	78.4↓	25.4↓	74.2	3.4	22.4↑	—	—	ND	ND	ND	One copy of the *HBB* gene	ND	ND	ND	SEA-HPFH, hete
25	42-M	5.19	120↓	74.2↓	23.2↓	83.7	7.2↑	9.1↑	—	—	ND	ND	ND	One copy of part of the *HBB* gene, hete	ND	ND	ND	Taiwanese Del, hete
26	25-F	3.76	117	92.5	31.2	86	1.8↓	2.4	—	—	ND	ND	ND	ND	ND	ND	ND	*HBD*: c.-127T > C, hete
27	33-M	4.71	149	95.2	31.5	97	1.3↓	1.7	—	—	ND	ND	ααα^anti3.7^/αα	ND	ND	ND	ααα^anti3.7^/αα	*HBD*: c.-127T > C, hete
28	23-F	4.31	114↓	81	26.6↓	96.7	3.3	—	—	—	ND	ND	ND	ND	ND	*HBB*: c.315 + 5G > C, hete	ND	*HBB*: c.315 + 5G > C, hete
29	25-F	4.55	111↓	80	24.4↓	86.5	2.4↓	—	—	—	ND	ND	ND	ND	ND	ND	ND	ND
30	39-F	4.64	103↓	73.5↓	22.3↓	97.9	2.1↓	—	—	—	ND	ND	ND	ND	ND	ND	ND	ND

F, female; M, male; RBC, red blood cell; Hb, hemoglobin; MCV, mean corpuscular volume; MCH, mean corpuscular hemoglobin; NT, not tested; ND, no variants have been detected; hete, heterozygote; homo, homozygote; Del, deletion; Dup, duplication.

a Nine SNPs that are not present in the reference *HBA2* gene, but are present in the *HBAP1* gene, of which the variant *HBA2*: c.*92A>G is pathogenic.

### SMRT sequencing accurately determines the genotype and chromosomal breakpoints of novel variants

#### Case 1: novel 32.8-kb deletion involving *HBA2* and *HBA1*

Case 1 presented with microcytic hypochromic anemia (MCV: 65.4 fL, MCH: 20.9 pg). HPLC was normal, but gap-PCR detected abnormal fragments in lengths of ∼1.7 and 1.4–1.7 kb. Sanger sequencing failed to detect any pathogenic variants in the *HBA* or *HBB* genes. MLPA suggested a heterozygous deletion spanning *HBZ*, *HBZP1*, *HBM*, *HBAP1*, *HBA2*, *HBA1*, and *HBQ1*, but breakpoints were unclear. SMRT sequencing confirmed the MLPA results and precisely mapped it to chr16:149,860–182,697 (GRCh38), spanning 32.8 kb (Figure 1b).

**Figure 1:**
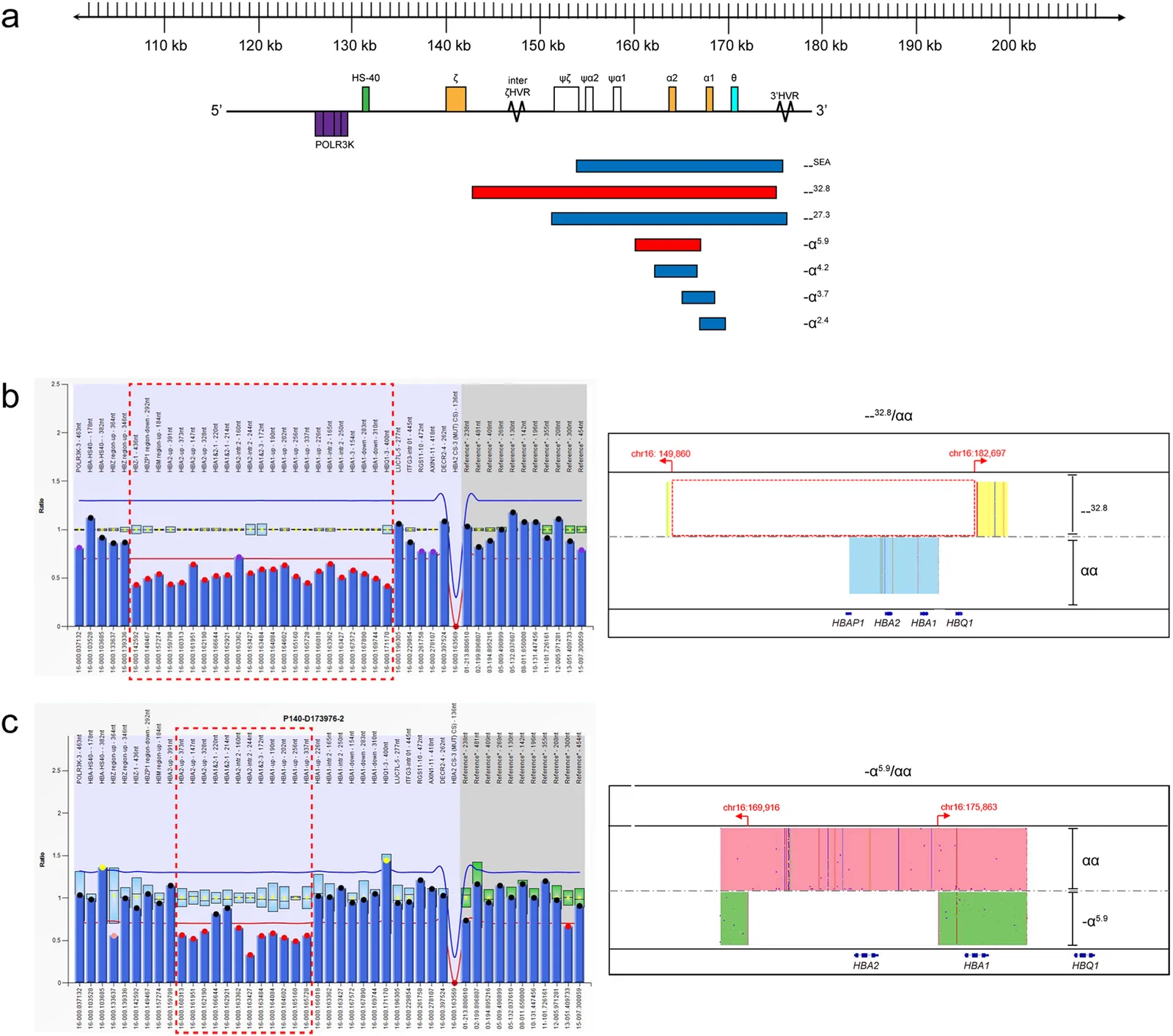
Variants in the α-globin gene cluster. **(a)** Schematic representation of seven deletions causing reduced expression of α-pearl proteins in this study. Colored boxes indicate regions of deletion, where blue boxes indicate reported deletions, red boxes indicate novel deletions. The length of the deletion fragments is in kb. **(b)** MLPA and SMRT sequencing of the novel 32.8-kb deletion including *HBA1* and *HBA2* genes. **(c)** MLPA and SMRT sequencing of the novel 5.9-kb deletion including the *HBA2* gene.

#### Case 2: novel 5.9-kb deletion including the *HBA2* gene

Although routine blood counts are normal (MCV: 87.6 fL, MCH: 28.8 pg) and Hb F is below the detection limit (<0.4%), the Hb A_2_ level is mildly reduced (2.3%). This genotype-phenotype profile is consistent with a silent α⁺-thalassemia carrier state. Gap-PCR showed two abnormal bands of 1.7 and >2.0 kb, suggesting an unknown/rare genetic anomaly. Sanger sequencing found no abnormalities. MLPA indicated heterozygous deletions in several *HBA* loci (*HBA2*-up, *HBA1* & Amp. 2–1, *HBA2*-intr.2, *HBA1* & Amp. 2–3, and *HBA1*-up probes), though breakpoints were undefined. SMRT sequencing confirmed the MLPA results, revealing a novel heterozygous deletion of approximately 5.9 kb (chr16:169,916–175,863, GRCh38). Given that the proband's wife carried the --^SEA^/αα genotype and was 22 weeks pregnant, there was a risk of Hb H disease in the fetus. Fortunately, prenatal diagnosis showed inheritance of only the 5.9-kb deletion, allowing the pregnancy to continue (Figure 1c).

#### Cases 3 and 4: novel 8.3-kb deletion including the *HBB* gene

Case 3 showed microcytic hypochromic anemia (MCV: 73.3 fL, MCH: 24.8 pg) with elevated Hb A_2_ (4.5%). Conventional testing only detected -α^3.7^/αα, prompting suspicion of an unidentified β-globin variant. Sanger sequencing was negative. MLPA detected a heterozygous *HBB* deletion, but breakpoints were undefined. SMRT sequencing confirmed the deletion in both the proband and her mother, with breakpoints at chr11:5,224,209–5,232,469 (GRCh38), totaling 8.3 kb (Figure 2b).

**Figure 2:**
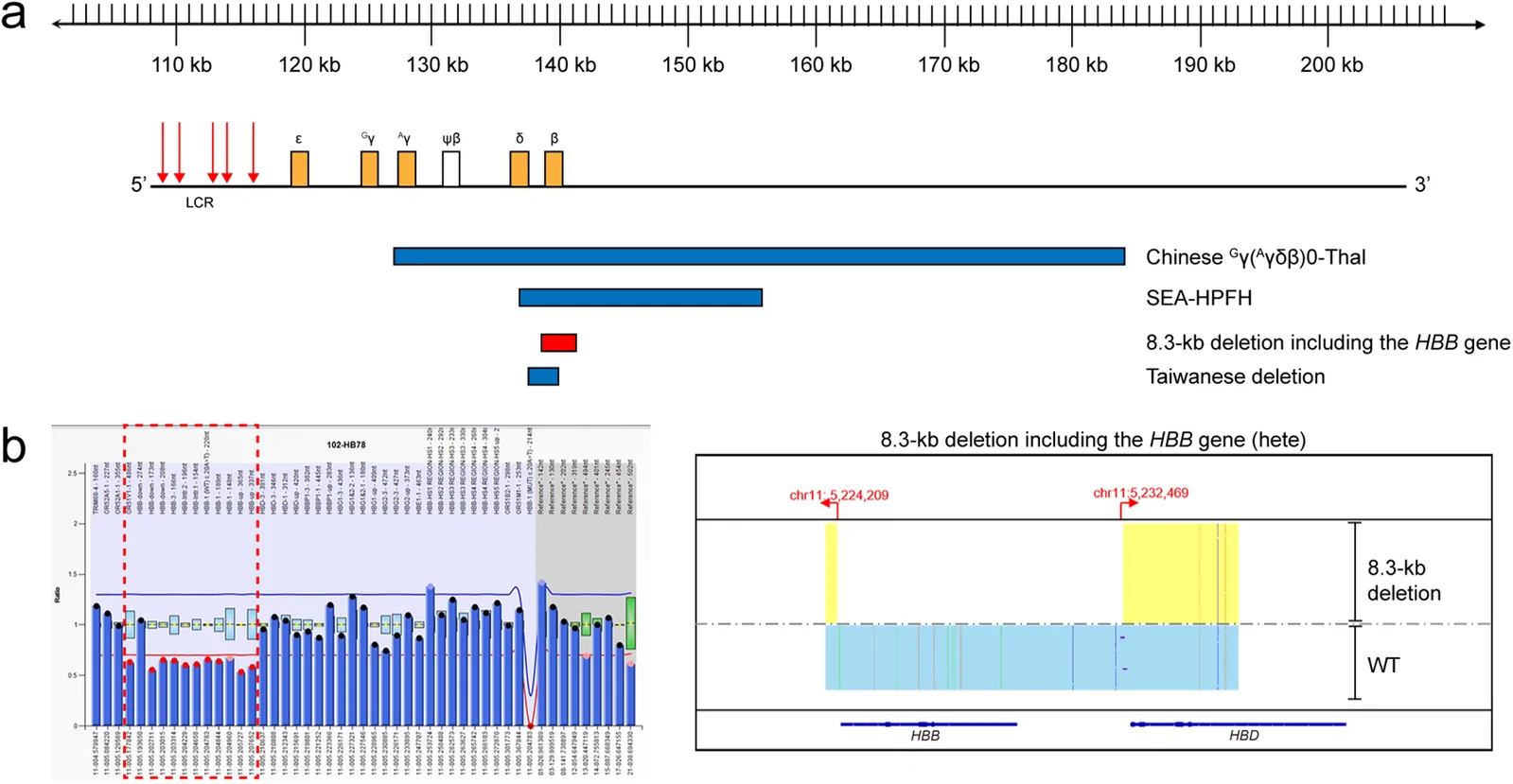
Variants in the β-globin gene cluster. **(a)** Schematic representation of five deletion variants causing decreased expression of β-pearl protein in this study. Colored boxes indicate regions of deletion, where blue boxes indicate reported deletions, the red box indicates the novel deletion. The length of the deletion fragments is in kb. **(b)** MLPA and SMRT sequencing of the novel 8.3-kb deletion including the *HBB* gene.

### SMRT sequencing accurately identifies complex structural variants

#### Cases 5–6: 7.4-kb duplication including the βδ-fusion gene (Hb anti-Lepore LiuZhou)

Two cases showed microcytic hypochromic anemia and elevated Hb A_2_ (16.5%, 14.2%), initially misdiagnosed as Hb E disease. MLPA showed triplicated *HBD*/*HBB* regions. SMRT sequencing confirmed the MLPA results and indicated the presence of a heterozygous duplication in the proband (Case 6) and his mother (Case 5). Bioinformatics analysis successfully localized the breakpoint at chr11:5,227,051–5,234,462 (GRCh38) and confirmed a duplication size of approximately 7.4 kb. This duplication included the βδ-fusion gene, which was finally determined as the Hb anti-Lepore Liuzhou (Figure 3a).

**Figure 3:**
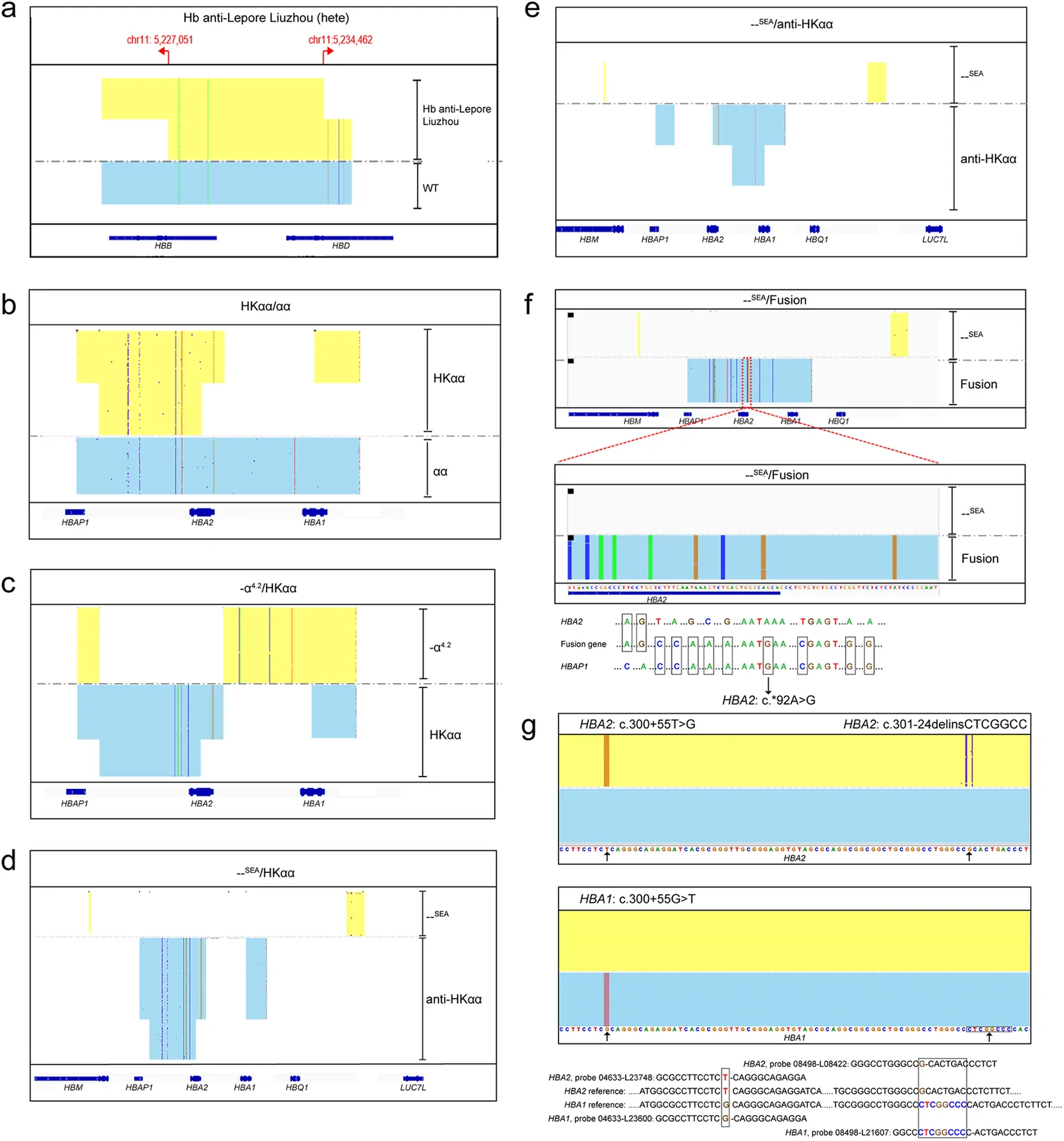
The complex structural variants identified in the α- and β-globin gene clusters. Shown are **(a)** Hb anti-Lepore LiuZhou, **(b)** HKαα/αα, **(c)** -α^4.2^/HKαα, **(d)** --^SEA^/HKαα, **(e)** --^SEA^/anti-HKαα, **(f)** α-fusion genes, and **(g)** α-globin patchwork alleles identified in our study.

#### Cases 7–12: complex α-globin rearrangements

In this study, cases 7 and 8 presented with decreased RBC and/or Hb, while the routine genetic analysis detected an α^+^-deletion (-α^3.7^). Case 9 only had a lower Hb A_2_ level of 2.2%, while the routine genetic analysis detected two α^+^-deletions (-α^3.7^ and -α^4.2^). Cases 10–12 had microcytic hypochromic anemia with normal/lower Hb A_2_ levels. However, the routine genetic analysis detected an α^0^-deletion (--^SEA^) and an α^+^-deletion (-α^3.7^ or -α^4.2^). Genotypes determined by the routine genetic analysis seemed more serious than predicted. MLPA identified α-deletions and α-triplications in these cases, with the phases unknown. SMRT found that α-triplications were all *in cis* with the α-deletions in these cases, determining the genotypes as HKαα or anti-HKαα, which could explain the phenotypes of these cases (Figures 3b–e).

#### Cases 13–15: *HBA* fusion genes

Three patients with rare Hb H disease were initially genotyped as --^SEA^/αα by Gap-PCR and RDB. This suggested the possible presence of an undetected variant in the α-globin gene cluster. Subsequent SMRT sequencing identified nine single-nucleotide substitutions in the 3′ untranslated region of *HBA2* (3′UTR) (Figure 3f), which included a pathogenic variant *HBA2*: c.*92A>G. BLAST analysis revealed high homology to the ψα1 pseudogene, suggesting a recombination event between the *HBA2* and *HBAP1* genes, which was designated as Fusion LZ (Figure 3g).

#### Cases 16–17: α-globin chimeric genes

MLPA results showed that there should be α-variants in cases 16 and 17. In case 16, one copy of the *HBA2*-intr.2 (probes 08498-L08422 and 04633-L23748) and three copies of the *HBA1*-intr.2 (probes 08498-L21607 and 08498-L21607) were identified. While in case 17, one copy of the *HBA1*-intr.2 (probe 04633-L23600) and three copies of the *HBA2*-intr.2 (probe 04633-L23748) were identified. According to the SMRT analyses, two *cis*-phased *HBA2* variants, c.300+55T>G and c.301–24delinsCTCGGCCC, and one *HBA1* variant c.300+55G>T were identified in the region where the MLPA probes bind (Figure 3g), leading to the recombination (α212 and α121) between the two homologous α-globin genes (Figure 3g).

### Correlation between hematological parameters and different thalassemia genotype variations

To evaluate the impact of different α-globin gene defect types on hematological parameters, 28 cases with genotypes confirmed by SMRT sequencing were divided into three groups based on their α-globin gene defect status: the α^0^-thalassemia heterozygote group (carrying --^32.8^, --^SEA^, or --^27.3^, *n* = 10), the α^+^-thalassemia heterozygote group (carrying -α^5.9^, -α^4.2^, -α^2.4^, or -α^3.7^, *n* = 4), and the group without α-globin gene defects (*n* = 14). Intergroup comparisons revealed that MCV and MCH levels in the α^0^-thalassemia heterozygote group were significantly lower than those in the other two groups, while no significant differences were observed between the α^+^-thalassemia heterozygote group and the non-α-defect group for these two parameters (Figure 4).

**Figure 4:**
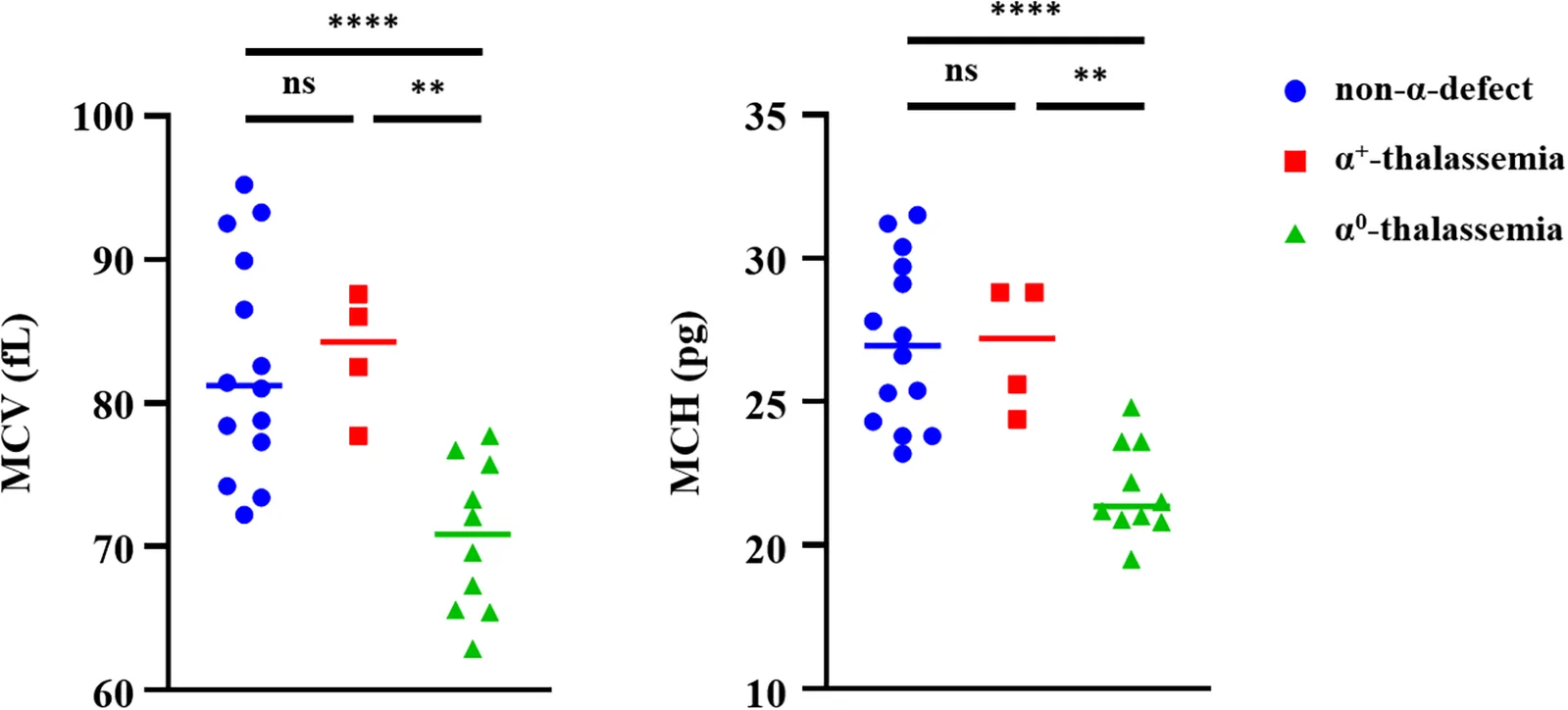
Comparison of hematological parameters among different α-globin gene defect groups. Scatter plots showing the distribution of mean corpuscular volume (MCV) and mean corpuscular hemoglobin (MCH) across the three groups stratified by α-globin gene defect status: the non-α-defect group (blue circle, *n* = 14), the α^+^-thalassemia heterozygote group (red square, *n* = 4), and α^0^-thalassemia heterozygote group (green triangle, *n* = 10). Intergroup comparisons were performed using one-way ANOVA. ****p < 0.0001; **p < 0.01; ns, not significant. Horizontal lines represent the mean.

### SMRT sequencing effectively detects rare thalassemia-related variants

In addition to the above variants, SMRT identified two α-deletions (-α^2.4^ and --^27.3^), one α-triplicates (ααα^anti3.7^), three β-deletions [Chinese ^G^γ(^A^γбβ)^0^-Thal, SEA-HPFH and Taiwanese deletion], one *HBB* variant (c.315+5G>C), and one *HBD* variant (c.-127T>C), which could explain subjects' clinical phenotypes. Subjects with three β-globin deletions showed microcytic hypopigmented anemia and elevated Hb F levels, especially with the SEA-HPFH variant (Table 1). One case with ααα^anti3.7^/αα and the *HBD* variant c.-127T>C showed a normal hematologic phenotype and reduced Hb A_2_, was misdiagnosed as αα/αα by conventional techniques.

## Discussion

### Detection of novel and complex variants by SMRT sequencing

In this study, conventional gap-PCR and PCR-RDB methods detected only three variants. In contrast, SMRT sequencing identified pathogenic variants in 28 cases, with results fully concordant with MLPA and Sanger sequencing. Notably, several rare or novel deletions were precisely characterized by SMRT sequencing, as detailed below. The novel 32.8-kb deletion (chr16:149,860–182,697) identified in case 1 represents a rare α^0^-thalassemia allele that removes the entire functional α-globin gene cluster. While similar in overall size to the previously reported --^CAL^ deletion (32.2 kb, chr16:147,601–179,801) (28, 29), the two deletions differ in both their proximal and distal breakpoints, with the novel deletion extending approximately 2.9 kb further toward the telomere. This extended region may encompass downstream regulatory elements, although its functional impact on the residual *HBQ1* gene or other flanking transcripts remains to be determined through experimental studies. The carrier presented with typical α^0^-thalassemia hematological features (MCV: 65.4 fL, MCH: 20.9 pg) and normal Hb A_2_ levels, a phenotype indistinguishable from that of common --^SEA^ heterozygotes (30). Clinically, when gap-PCR produces atypical bands and MLPA suggests a large deletion without precisely resolving the breakpoints, this novel deletion should be considered in the differential diagnosis, and SMRT sequencing can serve as an effective complementary tool for definitive characterization. The --^27.3^ deletion (chr16:158,664–185,974) affects a region and size similar to those of the common --^SEA^ deletion (NC_000016.10:g.165401_184701del), both spanning both functional α-globin genes. Heterozygotes carrying this deletion express only two functional α-globin genes and exhibit hematological features of α^0^-thalassemia, such as reduced MCV and MCH (31). The 5.9-kb deletion (-α^5.9^, chr16:169,916–175,863) is a rare α^+^-thalassemia allele that removes the *HBA2* gene while leaving *HBA1* intact. Heterozygotes carrying this deletion (Case 2) exhibit essentially normal red blood cell indices, apart from a mildly reduced Hb A_2_ level—a profile consistent with a silent α⁺-thalassemia carrier state. This observation suggests that, under steady-state conditions, a single functional *HBA1* gene is sufficient to sustain normal erythropoiesis. However, when this deletion is inherited in *trans* with an α^0^-thalassemia allele such as --^SEA^, the resulting compound heterozygous genotype (--^SEA^/-α^5.9^) would cause Hb H disease. In the family of Case 2, the proband (carrying -α^5.9^/αα) and his wife (carrying --^SEA^/αα) faced a 25% risk of conceiving a fetus with the --^SEA^/-α^5.9^ genotype. Prenatal diagnosis revealed that the fetus inherited only the paternal -α^5.9^ deletion without the maternal --^SEA^ allele (genotype: -α^5.9^/αα), thereby excluding the risk of Hb H disease and allowing the pregnancy to continue. This case underscores the clinical utility of SMRT sequencing in precisely defining deletion breakpoints, which enables targeted prenatal diagnosis and informed genetic counseling (27, 32). Accurate identification of such rare deletions is therefore critical for prenatal counseling. Notably, because its breakpoints differ from those of common α⁺-deletions (-α^3.7^ and -α^4.2^), the 5.9-kb deletion is not detectable by conventional gap-PCR (33). The gap-PCR electrophoresis results for this case showed bands of 1.7 kb and >2.0 kb, which were suggestive of an atypical deletion and subsequently resolved by SMRT sequencing. The -α^2.4^ deletion (NG_000006.1:g.36860_39252del) primarily involves *HBA1* and is located near the common -α^3.7^ deletion. Notably, the -α^2.4^ deletion, first reported in China (34), was also missed by conventional detection techniques, further underscoring the limitations of routine methods and the complementary value of SMRT sequencing for comprehensive variant detection.

Polyploidy in the α-globin gene cluster is relatively common. Misalignment during meiosis can result in unequal crossover events, leading to deletions (-α^3.7^ and -α^4.2^) and duplications (ααα^anti3.7^ and ααα^anti4.2^) (35, 36). Rare α-thalassemia genotypes, such as HKαα and anti-HKαα, involve hybrid fragments containing both a deletion and a triplication in *cis* on the same allele. Specifically, HKαα comprises -α^3.7^ plus ααα^anti4.2^, while anti-HKαα comprises -α^4.2^ plus ααα^anti3.7^ (37, 38). This configuration ultimately retains two functional α-globin genes (one *HBA2* and one *HBA1*), thereby explaining the mild or even normal hematological phenotype observed in carriers (e.g., cases 7–12). Conventional gap-PCR can only detect common α-deletions (--^SEA^, -α^3.7^, -α^4.2^, and --^THAI^). As a result, ααα^anti3.7^ and ααα^anti4.2^ duplications are misdiagnosed as wild type (αα/αα), while HKαα and anti-HKαα are mistaken for simple -α^3.7^ or -α^4.2^ heterozygotes—leading to inaccurate genotype interpretation and suboptimal genetic counseling (39). The frequency of HKαα exhibits significant regional variation; among Southeast Asian populations, its reported frequency among α-thalassemia carriers is 1%–3%, but the actual prevalence may be considerably underestimated due to this diagnostic masking (40). The clinical significance of HKαα/anti-HKαα extends beyond its own mild phenotype, particularly when co-inherited with β-thalassemia. Previous studies have demonstrated (40, 41) that each additional α-globin gene copy aggravates the α/β-globin chain imbalance, converting mild β-thalassemia traits into moderate or even transfusion-dependent thalassemia intermedia (non-transfusion-dependent thalassemia, NTDT) (42). In regions such as Guangxi, where both α- and β-thalassemia are highly prevalent, failure to identify HKαα/anti-HKαα may lead to systematic underestimation of disease severity in compound heterozygotes. SMRT sequencing, with its ability to resolve *cis*-phase configurations, provides definitive genotyping and accurate risk prediction, thereby offering critical evidence for clinical genetic counseling and prenatal diagnosis (32, 43, 44).

In Cases 13–15, conventional methods detected only a heterozygous --^SEA^ deletion, which alone cannot explain the observed Hb H disease phenotype. Our patients also carry a rare *HBA2*-*HBAP1* fusion allele, which may contribute to this phenotype (43, 45). The recently proposed Hb H disease severity scoring system (46) stratifies patients into mild, moderate, or severe categories based on seven clinical parameters. Given the presence of this rare fusion allele in our patients, the unique genetic background may modulate the clinical phenotype and therefore warrants individualized assessment to determine the precise severity of Hb H disease. SMRT sequencing further revealed that the 3′ UTR region of the *HBA2* gene harbored an extended sequence consisting of nine single-nucleotide substitutions that completely matched the corresponding region of the pseudogene *HBAP1* (ψα1), indicating the formation of an *HBA2-HBAP1* fusion gene. Among these nine substitutions, the known pathogenic variant is c.*92A>G, which disrupts the canonical polyadenylation signal (AATAAA → AATGAA), leading to reduced efficiency of mRNA cleavage and polyadenylation and consequently decreasing α-globin expression (47). The functional significance of the remaining eight substitutions—including the novel c.*136A>G identified exclusively in Guangxi cases—remains to be elucidated. Notably, the number of ψα1-derived substitutions in the fusion alleles exhibits a clear geographic gradient: Hunan cases carry 7 substitutions (47), Hainan (48) and Guangdong (49) cases carry 8 substitutions, while the three unrelated Guangxi cases in this study carry all 9 substitutions, with c.*136A>G being unique to Guangxi. Based on the observation that three unrelated Guangxi cases share an identical nine-substitution pattern, we hypothesize that this gradient pattern arose from a single ancestral fusion event, followed by the gradual accumulation of additional ψα1 sequences through gene conversion. However, this hypothesis requires further validation through haplotype-based population genetic studies. These findings are consistent with the notion that variant types across different regions of China are broadly similar, yet private lineage variants exist in specific populations (50). From a diagnostic perspective, such fusion alleles are undetectable by conventional gap-PCR or Sanger sequencing, and short-read NGS (150–300 bp) also struggles to accurately assign sequences to the *HBA2* gene due to the high homology between its 3′ UTR and that of *HBAP1*. Leveraging its full-length amplicon sequencing capability, SMRT sequencing can completely cover the *HBA2* gene and precisely identify fusion events and their breakpoints (43). Therefore, for any case in which only a single heterozygous α^0^-deletion (e.g., --^SEA^) is detected but the phenotype suggests Hb H disease, long-read sequencing is recommended to screen for occult α⁺-thalassemia alleles such as the *HBA2-HBAP1* fusion gene.

The coding regions (exons) of *HBA1* and *HBA2* are identical, and the second intron (IVS-II) differs by only two nucleotides (51). This high degree of homology renders the two genes susceptible to non-allelic homologous recombination, which can produce chimeric genes α212 and α121. In the present study, these chimeric genes were identified in cases 16 and 17, respectively. The formation of these chimeric genes involves only the interchange of the second intron sequence and does not alter the encoded α-globin amino acid sequence; hence, these chimeric genes are generally considered not to affect α-globin protein function (51). In clinical practice, individuals carrying these chimeric genes typically present with normal erythrocyte indices, with only a few studies reporting a mild decrease in Hb A_2_ percentage (52). Consequently, it is currently believed that these chimeric genes itself does not cause an α-thalassemia phenotype, although it may exert a very subtle effect at the expression level (52, 53). Our findings are consistent with this view: case 16 showed decreased Hb A_2_ (1.5%) and elevated Hb F (5.0%), while the case 17 exhibited borderline low MCV (78.8 fL) and MCH (25.3 pg). The latter abnormalities were attributable to a co-inherited *HBD* promoter variant (c.-127T>C) rather than the chimeric allele itself. Importantly, such rearrangements neither alter the copy number of functional α-globin genes nor directly cause an α-thalassemia phenotype, and therefore do not exacerbate the clinical severity of co-existing β-thalassemia or Hb H disease. From a diagnostic standpoint, these chimeric genes do not affect the primer binding sites for the common -α^3.7^ or -α^4.2^ deletions; consequently, these chimeric genes cannot be detected by conventional gap-PCR and are misclassified as wild type. Moreover, MLPA may misinterpret intronic nucleotide substitutions as deletions. Compounding these challenges, the high sequence homology between *HBA1* and *HBA2* makes it difficult for conventional sequencing software to accurately assign short reads to the correct gene, often leading to the misidentification of intronic differences as heterozygous variants of a single gene (54). By generating full-length reads, SMRT sequencing enables accurate discrimination between *HBA1* and *HBA2*, thereby avoiding misdiagnosis caused by these technical limitations (43, 55).

β-thalassemia is primarily caused by SNVs or small indels in the *HBB* gene, although large deletions also occur. Common large deletions in the Chinese population include Chinese ^G^γ(^A^γбβ)^0^-thal, SEA-HPFH, and Taiwanese deletion, all characterized by typical microcytic hypochromic anemia and elevated Hb F levels (56, 57), as seen in cases 20–25. The 8.3-kb deletion identified in cases 3 and 4 (hg38 chr11:5,224,209–5,232,469) encompasses the entire *HBB* coding region, resulting in a β^0^-thalassemia phenotype with elevated Hb A_2_ levels. Its clinical features resemble those of the Taiwanese deletion (58, 59), but with a notable intrafamilial difference: the mother has normal Hb F levels, while the daughter shows mildly elevated Hb F. This observation suggests the possible involvement of differential *trans*-acting modifiers; alternatively, the physiological decline of Hb F with age may partially account for this variation. Future functional studies—such as CRISPR-based modeling in erythroid cells—are needed to determine whether the deletion itself removes *cis*-regulatory elements involved in *HBG1*/*HBG2* silencing. Notably, the Taiwanese deletion (chr11:5,223,053–5,245,350) extends approximately 5 kb distally and may harbor additional regulatory sequences; the overlapping but not entirely concordant phenotypes suggest that the key determinants of Hb F elevation may reside within the commonly deleted region shared by both deletions. A 7.4-kb duplication (chr11:5,227,051–5,234,462) encompassing the *HBB* and *HBD* genes was identified in cases 5 and 6, and was previously designated as Hb anti-Lepore Liuzhou (26). Both cases exhibited elevated Hb A_2_ levels, and routine screening could easily misdiagnose them as Hb E. For both the 8.3-kb deletion and the 7.4-kb duplication, conventional methods—including gap-PCR, Sanger sequencing, and even MLPA—provide incomplete or misleading information. MLPA can confirm copy number changes but cannot pinpoint breakpoints (32, 60, 61); Sanger sequencing targeting only *HBB* exons completely misses the deletion; and routine hemoglobin analysis cannot distinguish anti-Lepore from Hb E. In contrast, SMRT sequencing, with its full-length coverage of the β-globin gene cluster, resolves these ambiguities and provides precise genotyping, underscoring its critical diagnostic value in atypical β-thalassemia phenotypes (15, 17, 32, 55).

### Comparison with conventional methods and clinical implications

While SMRT sequencing demonstrated a higher resolution in identifying complex structural variants, it is important to interpret these findings with clinical caution. In our cohort, MLPA provided diagnostic information in the majority of cases and is currently a mainstay in thalassemia screening due to its simplicity, cost-effectiveness, and widespread availability (62, 63). Although MLPA could not resolve exact breakpoints or novel fusion structures, it successfully indicated copy number changes in cases. These data remain informative for clinical decision-making in many routine cases. The primary advantage of SMRT sequencing lies in its ability to resolve diagnostically challenging cases, especially when conventional approaches yield inconclusive results. For instance, breakpoint-level information is useful in confirming carrier status in family studies, designing allele-specific PCR assays for prenatal diagnosis, and distinguishing pathogenic variants from benign duplications (44, 64, 65). Therefore, SMRT sequencing should not be viewed as a replacement for MLPA but rather as a complementary tool, particularly valuable in reference laboratories dealing with complex thalassemia genotypes (14, 32, 66).

### Comparison with short-read NGS

Due to its high throughput and ability to detect known and novel SNVs and small insertions/deletions, short-read NGS has been increasingly applied in thalassemia screening (12). However, the results of this study highlight three key limitations of short-read sequencing platforms, which SMRT sequencing successfully overcomes. First, resolution limitations in homologous regions. The coding sequences (CDS) of the *HBA1* and *HBA2* genes are identical, and they exhibit minor differences in the 5′ UTR and intronic regions. Short reads (150–300 bp) cannot span the distance between informative intronic variants and exons, making it difficult to determine the exact gene origin of a read. This limitation is further compounded by the presence of the pseudogene *HBAP1* (ψα1), whose 3′ UTR is nearly identical to that of *HBA2* (43, 67, 68). In this cohort, this limitation would have led to missed diagnoses in cases 13–15 (*HBA2-HBAP1* fusion gene) and cases 16–17 (chimeric genes), whereas SMRT sequencing successfully resolved all these complex variants. Second, limitations in detecting structural variants. Short-read NGS cannot reliably detect large deletions, duplications, or complex rearrangements with breakpoints spanning thousands of base pairs (16, 69–71). Even with specialized bioinformatics tools, MLPA and short-read sequencing can often only indicate copy number changes but fail to determine precise breakpoints or elucidate the genomic structure of rearrangements. The newly identified 32.8-kb, 5.9-kb, and 8.3-kb deletions in this study—all missed or only partially characterized by traditional methods—were fully resolved only through SMRT sequencing. Third, limitations of haplotype phasing. Short reads cannot determine whether multiple variants are located on the same chromosome (in *cis*) or on different chromosomes (in *trans*) (15, 67). This is especially critical for the HKαα and anti-HKαα alleles, as their deletions and duplications coexist in a *cis* configuration on the same allele; misclassifying such complex alleles as simple heterozygous deletions directly leads to inaccurate genetic counseling. By generating long reads spanning the entire α-globin gene cluster (>10 kb), SMRT sequencing achieved clear haplotype phasing for all HKαα/anti-HKαα cases in this cohort. In summary, although NGS remains an important tool for detecting SNVs and small insertions/deletions, its short read length imposes a fundamental limitation in the highly homologous and structurally complex globin gene cluster. SMRT sequencing overcomes these limitations through long-read sequencing and haplotype phasing analysis, enabling definitive identification of complex alleles—a capability of significant value for reference diagnosis in challenging thalassemia cases.

### Correlation between thalassemia genotype and hematological parameters

Based on SMRT sequencing genotyping results, this study identified 10 α^0^-thalassemia heterozygotes and 4 α^+^-thalassemia heterozygotes among the 28 carriers with confirmed genotypes. Intergroup comparisons of hematological parameters revealed that MCV and MCH levels in the α^0^ group were significantly lower than those in both the α^+^ group and the non-α-defect group, whereas no significant differences were observed between the α^+^ group and the non-α-defect group for these two parameters. These findings are highly consistent with the dose-dependent effect of α-globin gene expression: α-globin gene copy number directly determines the synthesis rate of α-globin chains, thereby regulating hemoglobin production and erythrocyte indices (72). When α-globin gene deletions reduce α-chain synthesis, the excess β-globin chains form unstable β-tetramers (Hb H), leading to premature destruction of erythroid precursors in the bone marrow (ineffective erythropoiesis) and ultimately manifesting as the classic microcytic hypochromic anemia phenotype (73–76). From a clinical diagnostic standpoint, this observation provides a quantitative basis for the preliminary differentiation between α^0^ and α^+^ carriers based on MCV and MCH levels—patients presenting with markedly reduced MCV and MCH are far more likely to be α^0^ carriers than α^+^ carriers. Clinicians may therefore prioritize α^0^-targeted molecular testing before proceeding to comprehensive sequencing, enabling a more efficient, stratified diagnostic workflow. In addition, this study identified two carriers with abnormally elevated Hb A_2_ (>10%) who carried the Hb anti-Lepore Liuzhou heterozygote, and carriers with markedly elevated Hb F (>20%) who carried the SEA-HPFH heterozygote. These observations suggest that when similar hematological parameters are encountered in clinical practice, these two rare pathogenic variants should be included in the differential diagnosis.

### Clinical utility, cost consideration, and limitations

From a clinical implementation standpoint, the adoption of SMRT sequencing in routine workflows remains constrained by its relatively high cost, long turnaround time, and the requirement for advanced bioinformatics infrastructure (18, 32, 77, 78). In contrast, MLPA offers a rapid, low-cost, and technically straightforward option suitable for large-scale screening, especially in resource-limited settings (79). We suggest that SMRT sequencing may serve as a second-tier test in cases with atypical hematological profiles, discordant genotype-phenotype results, or suspected complex rearrangements not explained by conventional methods (32, 43).

This study has several limitations. First, the sample size was limited to 30 cases, and the clinical significance of some novel findings requires further investigation through functional studies or familial segregation analysis. Second, cost-effectiveness analyses were not performed, and the direct clinical impact of defining exact breakpoints remains to be validated in longitudinal follow-up studies. Third, while SMRT sequencing improved diagnostic yield in our cohort, its broader clinical utility across diverse populations needs further assessment. Fourth, the novel deletions identified by SMRT sequencing (32.8-kb, 5.9-kb, and 8.3-kb deletions) were not independently validated by breakpoint PCR. Although SMRT sequencing provides single-molecule resolution and enables precise breakpoint characterization, independent PCR validation would further strengthen the reliability of these findings. All of the above limitations should be addressed in future studies.

## Conclusions

In conclusion, SMRT sequencing improves the molecular characterization of complex thalassemia cases, particularly when traditional methods are inconclusive. While not intended to replace routine diagnostics, it serves as a powerful complementary tool in clinically ambiguous or genetically atypical cases. As sequencing technology becomes more accessible, its broader application in clinical diagnosis warrants further investigation.

## Data Availability

The original contributions presented in the study are publicly available. The raw SMRT sequencing data have been deposited in the Genome Sequence Archive 80, 81 (GSA-Human) (https://ngdc.cncb.ac.cn/gsa-human) under accession number HRA020430. The same datasets have been deposited in the ITHANTET database (https://www.ithanet.eu/community/experts?expID=249), where the novel deletions identified in this study are available under accession numbers IthaID: 3963 (32.8-kb deletion), IthaID: 3966 (5.9-kb deletion), and IthaID: 3958 (8.3-kb deletion).
